# Improved glycerol utilization by a triacylglycerol-producing *Rhodococcus opacus* strain for renewable fuels

**DOI:** 10.1186/s13068-015-0209-z

**Published:** 2015-02-26

**Authors:** Kazuhiko Kurosawa, Andreas Radek, Jens K Plassmeier, Anthony J Sinskey

**Affiliations:** Department of Biology, Massachusetts Institute of Technology, 77 Massachusetts Avenue, Cambridge, MA 02139 USA; Engineering Systems Division, Massachusetts Institute of Technology, 77 Massachusetts Avenue, Cambridge, MA 02139 USA; Present address: Institute of Bio- and Geosciences, IBG-1: Biotechnology, Systems Biotechnology, Forschungszentrum Juelich, 52425 Juelich, Germany

**Keywords:** Triacylglycerol, Renewable fuels, *Rhodococcus opacus*, Adaptive evolution, Glycerol utilization, Co-fermentation

## Abstract

**Background:**

Glycerol generated during renewable fuel production processes is potentially an attractive substrate for the production of value-added materials by fermentation. An engineered strain MITXM-61 of the oleaginous bacterium *Rhodococcus opacus* produces large amounts of intracellular triacylglycerols (TAGs) for lipid-based biofuels on high concentrations of glucose and xylose. However, on glycerol medium, MITXM-61 does not produce TAGs and grows poorly. The aim of the present work was to construct a TAG-producing *R. opacus* strain capable of high-cell-density cultivation at high glycerol concentrations.

**Results:**

An adaptive evolution strategy was applied to improve the conversion of glycerol to TAGs in *R. opacu*s MITXM-61. An evolved strain, MITGM-173, grown on a defined medium with 16 g L^−1^ glycerol, produced 2.3 g L^−1^ of TAGs, corresponding to 40.4% of the cell dry weight (CDW) and 0.144 g g^−1^ of TAG yield per glycerol consumed. MITGM-173 was able to grow on high concentrations (greater than 150 g L^−1^) of glycerol. Cultivated in a medium containing an initial concentration of 20 g L^−1^ glycerol, 40 g L^−1^ glucose, and 40 g L^−1^ xylose, MITGM-173 was capable of simultaneously consuming the mixed substrates and yielding 13.6 g L^−1^ of TAGs, representing 51.2% of the CDM. In addition, when 20 g L^−1^ glycerol was pulse-loaded into the culture with 40 g L^−1^ glucose and 40 g L^−1^ xylose at the stationary growth phase, MITGM-173 produced 14.3 g L^−1^ of TAGs corresponding to 51.1% of the CDW although residual glycerol in the culture was observed. The addition of 20 g L^−1^ glycerol in the glucose/xylose mix resulted in a TAG yield per glycerol consumed of 0.170 g g^−1^ on the initial addition and 0.279 g g^−1^ on the pulse addition of glycerol.

**Conclusion:**

We have generated a TAG-producing *R. opacus* MITGM-173 strain that shows significantly improved glycerol utilization in comparison to the parental strain. The present study demonstrates that the evolved *R. opacus* strain shows significant promise for developing a cost-effective bioprocess to generate advanced renewable fuels from mixed sugar feedstocks supplemented with glycerol.

**Electronic supplementary material:**

The online version of this article (doi:10.1186/s13068-015-0209-z) contains supplementary material, which is available to authorized users.

## Background

The development of upgrading technologies capable of producing substitutes for petroleum-based fuels and chemicals has gained momentum [1-3]. Glycerol is an inevitable by-product generated during biodiesel and bioethanol production processes [4-7]. In fact, in the biodiesel production process, glycerol is the main by-product—approximately 10% (*w*/*w*) by weight of the total product—of the transesterification of triacylglycerols from animal fats and plant oils with an alcohol [6]. Bioethanol produced through the fermentation of sugars by yeasts is also accompanied by the generation of glycerol as a fermentation by-product, comprising up to 2% by volume of the liquid fraction in the whole stillage [8]. The utilization of glycerol as a carbon source for microbial production processes has not been studied extensively, because glycerol was more expensive than other carbon sources used in the conventional fermentation industry. On the contrary, methods for producing glycerol by fermentation had been studied [9]. However, due to the dramatic increase in renewable fuel productions worldwide over the last few years, glycerol production has increased as a waste product, which has led to a decrease in its price, thereby allowing it to become an attractive feedstock for production of value-added materials by fermentation [4,6].

In microorganisms, glycerol metabolism has been better understood in *Escherichia coli* [10]. Although *E. coli* was long thought to require the presence of external electron acceptors (respiratory metabolism) for glycerol utilization, it has recently been shown that the bacterium can metabolize glycerol in a fermentative manner (in the absence of electron acceptors) [11]. Glycerol dissimilation in *E. coli* can proceed through three different routes to produce the glycolytic intermediate dihydroxyacetone phosphate (DHAP): the aerobic GlpK (glycerol kinase encoded by *glpK*)-GlpD (aerobic glycerol-3-phosphate dehydrogenase encoded by *glpD*) and anaerobic GlpK (glycerol kinase encoded by *glpK*)-GlpABC (anaerobic glycerol-3-phosphate dehydrogenase encoded by *glpABC*) respiratory routes, or the GldA (glycerol dehydrogenase encoded by *gldA*)-DhaKLM (dihydroxyacetone kinase encoded by *dhaKLM*) fermentative route [12-14]. Of these routes, the GldA-DhaKLM fermentive route has been shown to enable efficient utilization of glycerol under both anaerobic and microaerobic conditions. The glycerol metabolism is regulated in various ways. In particular, in the presence of glycerol with glucose, wild-type *E. coli* has been known to exhibit diauxic growth, showing that glucose is preferentially consumed before glycerol [15,16]. While many microorganisms have been shown to ferment glycerol, the fermentative metabolism of glycerol has been reported only in species of the genera *Anaerobiospirillum* [17], *Bacillus* [18], *Citrobacter* [18], *Clostridium* [19], *Enterobacter* [20], *Escherichia* [11], *Klebsiella* [21], *Lactobacillus* [18], and *Propionibacterium* [22]. More recently, there have been extensive studies for the development of microbiological processes to convert glycerol into various value-added materials, aimed at the production of arabitol [23], 2,3-butanediol [24], butanol [25], citric acid [26], ethanol [27,28], hydrogen [29,30], lactic acid [31], polyhydroxybutyrate [32], 1,2-propanediol [33], 1,3-propanediol [34], propionic acid [35], succinate [14], and triacylglycerols (TAGs) [36,37].

TAGs are esters in which three molecules of fatty acids are linked to glycerol and exploited as the precursor to produce lipid-based biofuels such as biodiesel and hydrocarbon fuels [38,39]. TAGs are found extensively as the carbon storage molecule in animals, plants, algae, and microorganisms [40,41]. Many sources of TAGs, with the exception of those with very short chain fatty acids, are converted to hydrocarbon-based biofuels that are identical in virtually every respect to commercially available petroleum-derived fuels [42,43]. Practically, renewable jet fuel, termed hydroprocessed esters and fatty acids (HEFA), made from vegetable-based TAG-containing feedstocks, has been successfully tested in military and commercial aircrafts [44,45]. However, technologies for cost-effectively converting renewable natural resources to biofuel molecules, in order to minimize the conflict between food and fuel use, have not yet been developed [46]. Oleaginous microorganisms that utilize a great variety of substrates offer benefits for TAG production from biological resources such as waste glycerol and lignocellulosic biomass [47,48].

*Rhodococcus opacus* PD630 produces TAGs consisting primarily of C16 and C18 series of long chain fatty acids, which are quite similar to those of vegetable-derived TAGs [49]. Steinbüchel and coworkers [50] demonstrated that *R. opacus* PD630 grown on gluconate is capable of accumulating up to 76% of the cell dry weight (CDW) as TAGs. We have reported that *R. opacus* PD630 has a rare ability to produce large amounts of TAGs when grown in the presence of high concentrations of glucose [51,52]. In addition, we recently engineered xylose fermenting strains that are capable of completely and simultaneously utilizing both xylose and glucose to produce large amounts of TAGs in the presence of high sugar concentrations [53,54]. Strain MITXM-61 grown in corn stover hydrolysate containing 118 g L^−1^ of initial sugars was capable of completely utilizing both xylose and glucose in the genuine lignocellulosic feedstock and yielded 15.9 g L^−1^ of TAGs with a productivity of 0.133 g L^−1^ h^−1^, corresponding to 54% of the CDW [54]. However, the strain does not produce TAGs on glycerol, and the growth is poor. Here, we aimed to improve glycerol utilization in *R. opacus* to ensure the effective use of glycerol and constructed a TAG-producing *R. opacus* strain capable of high-cell-density cultivation at high concentrations of glycerol or mixtures of glucose/xylose/glycerol.

## Results

### Construction of a robust glycerol-fermenting *R. opacus* strain

The cell growth of *R. opacus* PD630 was extremely poor in glycerol fermentations. In order to generate a TAG-producing strain with improved growth on glycerol, we sought to apply an adaptive evolution approach. Competent cells of the engineered xylose-fermenting *R. opacus* MITXM-61 strain, a PD630 derivative strain, were treated by electroporation and spread on a defined agar medium containing 16 g L^−1^ glycerol as the sole carbon source. Several colonies appeared on the plates, and the clones were isolated after 10 days of cultivation (Additional file 1: Figure S1). The growth of five isolates, termed MITGM-71, −72, −73, −74, and −81, was tested in a defined medium with 16 g L^−1^ glycerol in flasks. The cell growth of those strains started after a long lag phase that lasted from 3 to 4 days, as shown in Additional file 1: Figure S2. Strain MITGM-73 exhibited robust cell growth, reaching an optical density (OD_660_) of approximately 10 after 6 days of cultivation, but exhibited very slow growth on glycerol compared to other carbon sources. To further improve its glycerol utilization, MITGM-73 was subjected to an adaptive evolution procedure to select fast-growing glycerol-fermenting variants. Sequential transfers of MITGM-73 cells in batch flask cultivations with defined medium, supplemented 100 g L^−1^ glycerol, were carried out. Following inoculation, the culture was allowed to grow until the early stationary phase and then inoculated into the fresh medium. This transfer procedure was repeated for four iterations. The cell growth during the adaptive evolution is presented in Additional file 1: Figure S3. The initial culture grew slowly with a lag phase of 3 days, but after one transfer, it eventually grew faster with a short lag phase of 1 day. After five batch cultures, the culture was streaked on the plates with glycerol for isolated colonies. One of the fastest growing isolates was selected and designated strain MITGM-173 after comparing 20 isolates for their growth in the glycerol medium.

### Growth of *R. opacus* MITGM-173 with high glycerol concentrations

**Figure 1:**
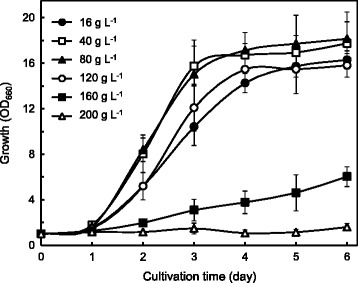
**Growth of** ***R. opacus*** **MITGM-173 on varying concentrations of glycerol.** Glycerol concentrations of defined media were 16, 40, 80, 120, 160, and 200 g L^−1^ in shake flasks. Values and error bars represent the mean and s.d. of triplicate experiments.

Our previous studies [51,54] showed that *R. opacus* PD630 derivatives were capable of growing on glucose and xylose at high initial concentrations of greater than 200 g L^−1^ as the sole carbon source. We examined the growth of strain MITGM-173 on defined media with initial glycerol concentrations of 16, 40, 80, 120, 160, or 200 g L^−1^ in flask cultures (Figure 1). Growth of the strain began after 2 days of cultivation in media containing up to 160 g L^−1^ of glycerol, and the culture reached stationary phase after 5 days of cultivation on concentrations of 16, 40, 80, and 120 g L^−1^ glycerol.

### TAG production of *R. opacus* MITGM-173 on glycerol and/or glucose

**Figure 2:**
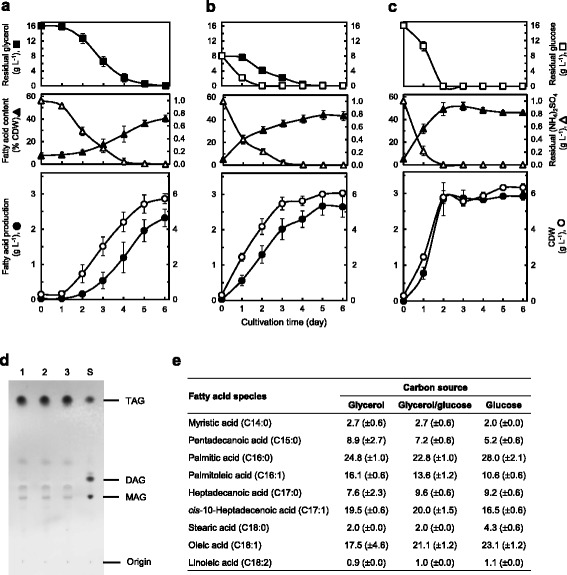
**TAG production from glycerol and/or glucose by** ***R. opacus*** **MITGM-173. (a-c)** Time course kinetics of TAG production as fatty acids. The strain was grown in defined media containing 16 g L^−1^ glycerol **(a)**, a mixture of 8 g L^−1^ glycerol and 8 g L^−1^ glucose **(b)**, and 16 g L^−1^ glucose **(c)** in shake flasks. Values and error bars represent the mean and s.d. of triplicate experiments. **(d)** Thin-layer chromatography analysis of the crude organic extracts obtained from the cells grown on glycerol **(a)**, glycerol/glucose **(b)**, and glucose **(c)** for 6 days. Lipids were extracted and separated on a silica gel plate as described in the “Methods” section. Lipid standards of TAG (1,2-dioleoyl-3-stearoyl-*rac*-glycerol), DAG (1,2-dipalmitoyl-*rac*-glycerol), and MAG (DL-α-palmitin) were used to identify the Rf value for TAG under the conditions used. Lanes: 1, crude lipid extract (10 μg) on glycerol; 2, crude lipid extract (10 μg) on glycerol/glucose; 3, crude lipid extract (10 μg) on glucose; S, TAG (3 μg)/DAG (3 μg)/MAG (3 μg) mixtures. **(e)** Fatty acid composition as percentage of total fatty acids (g g^−1^) of lipids from the cells growing in the defined medium containing glycerol **(a)**, glycerol/glucose **(b)**, or glucose **(c)** for 6 days. Data are results of triplicate experiments, ±s.d.

To elucidate the glycerol-assimilation profile by strain MITGM-173, we carried out flask cultivations in defined media containing either 16 g L^−1^ glycerol, a mixture of 8 g L^−1^ glycerol and 8 g L^−1^ glucose, or 16 g L^−1^ glucose. In these cultures, the kinetics of TAG production as fatty acids, CDW, fatty acid content as percent of CDW, and residual sugars and (NH_4_)_2_SO_4_ present in culture supernatants were determined (Figure 2a–c). When MITGM-173 was cultivated on glycerol alone (Figure 2a), the growth began after 1 day of cultivation and TAG accumulation increased after (NH_4_)_2_SO_4_ was depleted. In these cultures, maximum TAG production of 2.3 (±0.3) g L^−1^, representing 40.4 (±3.1) % of CDW occurred after 6 days of cultivation, at which point the residual glycerol was completely consumed. Growing on a glycerol/glucose mix (Figure 2b) and glucose alone (Figure 2c), resulted in TAG production of 2.7 (±0.3) g L^−1^ and 2.9 (±0.2) g L^−1^, respectively, corresponding to yields of 44.4 (±3.9) % and 47.8 (±3.6) % of CDW, respectively, during the stationary phase, which was 5 and 3 days post-inoculation, respectively. MITGM-173 grown on a glycerol/glucose mix had a short lag phase and a fast generation time in comparison with that on glycerol alone. The concentrations of glycerol and glucose in the medium simultaneously decreased over time although utilization of glycerol was delayed in the first day, and the complete consumption of glycerol and glucose occurred after 4 and 2 days of cultivation, respectively. During 6 days of cultivation, the maximum yield of TAG per gram of carbon source consumed was 0.144 (±0.015) g g^−1^ on glycerol alone, 0.169 (±0.018) g g^−1^ on a glycerol/glucose mix, and 0.181 (±0.002) g g^−1^ on glucose alone, respectively. When a mixture of glycerol and glucose was used as the carbon substrates in the medium, both the maximum TAG production and TAG yield per carbon source consumed were lower than those attained with glucose alone but higher than those with glycerol alone. The identity of the lipids and the fatty acid composition profiles of MITGM-173 cells grown under these conditions were quite similar to one another. A thin-layer chromatography (TLC) analysis of the crude organic extract from the cells showed that the TAG fraction amounted to approximately 90% (*w*/*w*) of the extractable lipids (Figure 2d). The fatty acid composition of the lipids was composed mainly of palmitic acid (22% to 28%), *cis*-10-heptadecenoic acid (16% to 20%), and oleic acid (17% to 24%), as revealed by gas chromatography (GC) analysis (Figure 2e).

Meanwhile, cultivating in defined media containing either 16 g L^−1^glycerol, a mixture of 8 g L^−1^ glycerol and 8 g L^−1^ glucose, or 16 g L^−1^ glucose, the parental strain MITXM-61 was hardly able to grow on glycerol alone (Additional file 1: Figure S4a). In the case of culturing on glucose alone (Additional file 1: Figure S4c) and mixed glycerol/glucose substrates (Additional file 1: Figure S4b), the TAG production in the stationary phase 3 days post-inoculation, at which point the residual glucose was completely consumed, was 2.9 (±0.2) g L^−1^ and 1.0 (±0.2) g L^−1^, respectively, corresponding to 48.9 (±1.9) % CDW and 30.4 (±2.9) % CDW, respectively. MITXM-61 was also unable to utilize glycerol in the mixed substrates when glycerol was included in the medium together with glucose. The results demonstrated that the evolved strain MITGM-173 harbored significantly improved glycerol-utilizing capacity, compared to the parental strain MITXM-61.

### Optimization of TAG production from glycerol by *R. opacus* MITGM-173

High-cell-density cultivation is essential to maximize volumetric productivity and to reduce production costs, and the media used should be composed of highly concentrated carbon sources [55,56]. We have demonstrated that TAG production in *R. opacus* is greatly affected by the ratio of carbon to nitrogen (C/N) in the medium [51-54]. The operational C/N ratio of glycerol and (NH_4_)_2_SO_4_ in a defined medium for maximum production of TAGs by strain MITGM-173 was optimized using a response surface methodology. The experimental design model assigned nine combinations of glycerol and (NH_4_)_2_SO_4_ concentrations including three repetitions at a central point (100 g L^−1^ glycerol and 6.23 g L^−1^ (NH_4_)_2_SO_4_) for a total of 11 bioreactors in batch fermentations. The design matrix of the variables in coded units and actual concentrations along with the experimental response is presented in Table 1. Strain MITGM-173 grown in the medium containing 100 g L^−1^ glycerol and 6.23 g L^−1^ (NH_4_)_2_SO_4_ yielded maximum TAGs of 13.8 (±0.6) g L^−1^, representing 45.2 (±0.7) % of the CDW, at which point the residual glycerol was almost completely consumed. Strain MITGM-173 had a critical feature capable of performing high-cell-density cultivation at high glycerol concentrations and producing large amounts of TAGs. We performed analysis on the experimental data using the software StatGraphics. The coefficient of determination (*R*^2^) was 0.9314, suggesting a relatively high correlation between predicted and experimental values. The estimated parameters from simulation with the model equation are as follows:

$$ Y = -8.66238 + 0.246034{X}_1 + 3.26793{X}_2\ \hbox{--}\ 0.0013664{X_1}^2 + 0.00250372{X}_1{X}_2\ \hbox{--}\ 0.267951{X_2}^2 $$

**Table 1:** **Central composite experimental design matrix defining glycerol and (NH**_4_ **)**_2_ **SO**_4_ **concentrations**

**Run**	**Real values**		**Coded values**		**Fatty acid production**	
	***X***_1_	***X***_2_	***X***_1_	***X***_2_	**% CDW**	**g L** ^**−1**^ **of culture**
1	100	6.23	0	0	45.9	13.9
2	30	1.83	−1	−1	44.3	5.1
3	100	6.23	0	0	45.2	14.3
4	200	6.23	1.41	0	6.0	0.2
5	30	10.63	−1	1	18.2	2.1
6	100	6.23	0	0	44.5	13.2
7	170	10.63	1	1	10.8	0.5
8	170	1.83	1	−1	14.6	0.4
9	0	6.23	−1.41	0	0.0	0.0
10	100	12.45	0	1.41	23.0	6.5
11	100	0	0	−1.41	0.0	0.0

*R. opacus* MITGM-173 was inoculated in the modified defined media in bioreactors. The data for TAG production as fatty acids represent the maximum values during 10 days of cultivation.

**Figure 3:**
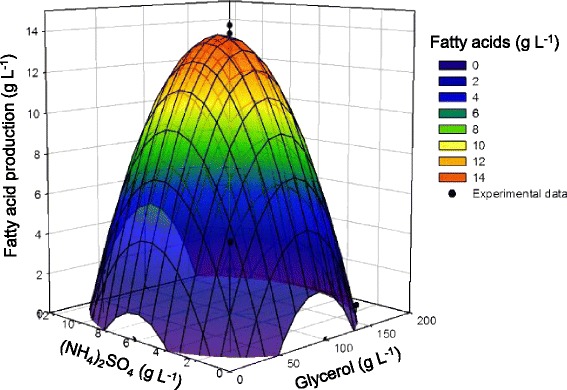
**Response surface plot of the effect of glycerol and (NH**_4_ **)**_2_ **SO**_4_ **concentrations on TAG production.** As fatty acids by *R. opacus* MITGM-173. Curves and points represent predicted values and experimental data, respectively.

**Figure 4:**
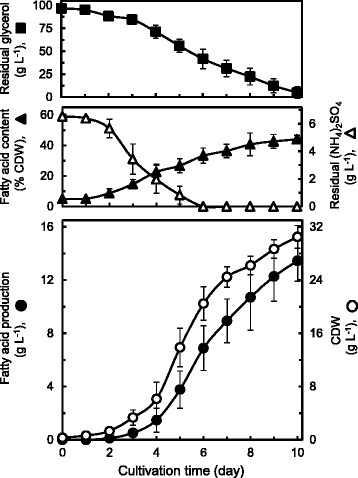
**Time course of TAG production as fatty acids from glycerol by** ***R. opacus*** **MITGM-173.** Performed under the optimized conditions. The strain was grown in a modified defined medium containing 96 g L^−1^ glucose and 6.55 g L^−1^ (NH_4_)_2_SO_4_ in bioreactors. Values and error bars represent the mean and s.d. of triplicate experiments.

*X*_1_ glycerol concentration (g L^−1^), *X*_2_ (NH_4_)_2_SO_4_ concentration (g L^−1^).

where *Y* is the predicted response (TAG production, g L^−1^ as fatty acids) and *X*_1_ and *X*_2_ are coded values of glycerol concentration (g L^−1^) and (NH_4_)_2_SO_4_ concentration (g L^−1^), respectively. The surface plots illustrated by the equation are presented (Figure 3). The experimental design predicted that growing MITGM-173 cells in a defined medium with a C/N ratio of 14.7 containing 96.0 g L^−1^ glycerol and 6.55 g L^−1^ (NH_4_)_2_SO_4_ would result in a maximal TAG production of 13.8 g L^−1^ as fatty acids. The C/N ratio for maximum TAG production of MITGM-173 on glycerol was slightly lower than that (C/N of 17.8) of PD630 on glucose [51] and that (C/N of 16.5) of MITXM-61 on xylose [54]. The predicted yield was validated by batch-culture fermentations with the optimized conditions. As predicted, the maximum TAG production of 13.4 (±1.5) g L^−1^ as fatty acids corresponding to 44.0 (±2.6) % of the CDW occurred after 10 days of cultivation, which was close to the predicted yield (Figure 4). Under these cultural conditions, the yield of total fatty acids per gram of glycerol consumed was 0.147 (±0.007) g g^−1^.

### Batch fermentation of *R. opacus* MITGM-173 on mixtures of glucose and xylose with glycerol

**Figure 5:**
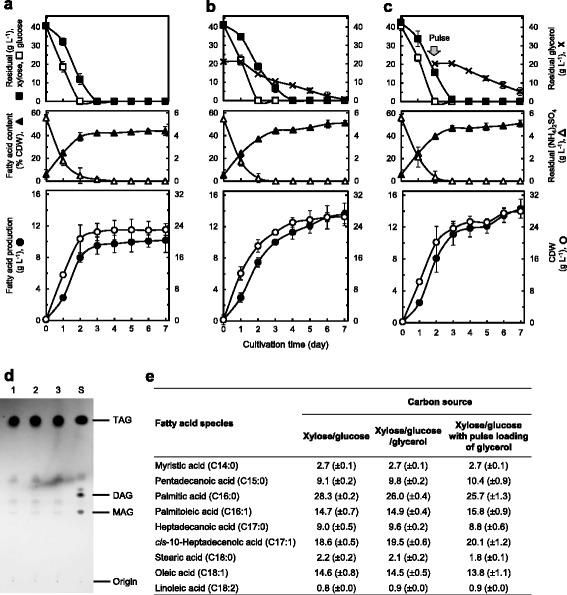
**TAG production from mixed substrates of glucose, xylose, and glycerol by** ***R. opacus*** **MITGM-173. (a-c)** Time course kinetics of TAG production as fatty acids. The strain was grown in modified defined media supplemented with 5.56 g L^−1^ (NH_4_)_2_SO_4_ containing a mixture of 40 g L^−1^ xylose and 40 g L^−1^ glucose **(a)**, a mixture of 40 g L^−1^ xylose, 40 g L^−1^ glucose and 20 g L^−1^ glycerol **(b)**, and a mixture of 40 g L^−1^ xylose and 40 g L^−1^ glucose with pulse loading of 20 g L^−1^ glycerol after 2 days of cultivation **(c)** in bioreactors. Values and error bars represent the mean and s.d. of triplicate experiments. **(d)** Thin-layer chromatography analysis of the crude organic extracts from the cells growing on xylose/glucose **(a)**, xylose/glucose/glycerol **(b)**, and xylose/glucose with pulse loading of glycerol **(c)** for 7 days. Lanes: 1, crude lipid extract (10 μg) on xylose/glucose; 2, crude lipid extract (10 μg) on xylose/glucose/glycerol; 3, crude lipid extract (10 μg) on xylose/glucose with pulse loading of glycerol; S, TAG (6 μg)/DAG (3 μg)/MAG (3 μg) mixtures. **(e)** Fatty acid composition as percentage of total fatty acids (g g^−1^) of lipids from the cells growing in the defined medium containing xylose/glucose **(a)**, xylose/glucose/glycerol **(b)**, or xylose/glucose with pulse loading of glycerol **(c)** for 7 days. Data are results of triplicate experiments, ±s.d.

We next explored the impact of TAG production by *R. opacus* MITGM-173 on mixtures of xylose and glucose, supplemented with glycerol, in the bioreactor system. Lignocellulosic hydrolysates are mixtures of hexoses and pentoses, mostly glucose and xylose with a typical mass ration around 2:1 [57]. Glycerol is produced as a by-product at levels of approximately 10% (*w*/*w*) of the total biodiesel generated [4-7]. Consequently, we investigated TAG production of strain MITGM-173 on mixed carbon sources in a 2:2:1 ratio of glucose/xylose/glycerol with an initial total substrate concentration of 100 g L^−1^. When cells of MITGM-173 were inoculated into a modified defined medium containing initial concentrations of 40 g L^−1^ xylose and 40 g L^−1^ glucose (Figure 5a), the cell growth increased rapidly after 12 h of cultivation, and nitrogen depletion occurred between 2 and 3 days. Glucose was completely depleted after 2 days and xylose after 3 days as a result of the concomitant consumption of xylose and glucose in the medium. The TAG production as fatty acids after 3 and 7 days of cultivation was 9.5 (±1.1) g L^−1^ and 10.2 (±1.5) g L^−1^, respectively, corresponding to 42.3 (±0.8) % and 44.0 (±3.9) %, respectively, of the CDW. The maximum yield of TAGs as fatty acids per gram of carbon source consumed was 0.128 (±0.009) g g^−1^ during 7 days of cultivation. In contrast, when MITGM-173 was cultivated in media containing initial concentrations of 40 g L^−1^ xylose, 40 g L^−1^ glucose, and 20 g L^−1^ glycerol (Figure 5b), and initial concentrations of 40 g L^−1^ xylose and 40 g L^−1^ glucose with pulse loading of 20 g L^−1^ glycerol after 2 days of cultivation (Figure 5c) and the TAG production after 3 and 7 days of cultivation were 10.0 (±0.1) g L^−1^ and 13.6 (±1.4) g L^−1^, respectively, corresponding to 44.4 (±1.4) % CDW and 51.2 (±2.1) % CDW, respectively, on the xylose/glucose/glycerol mix, and 11.1 (±1.4) g L^−1^ and 14.3 (±1.2) g L^−1^, respectively, corresponding to 39.8 (±0.6) % CDW and 51.1 (±3.1) % CDW, respectively, on the xylose/glucose mix with the pulse loading glycerol. The concentrations of all three carbon sources in the media simultaneously decreased over time, although the consumption of glycerol was delayed in the first day after the addition of glycerol as compared to that of glucose or xylose. The consumption of xylose, glucose and glycerol in the xylose/glucose/glycerol mix was complete after 4, 2 and 7 days, respectively, of cultivation (Figure 5b). When the glycerol pulse was applied to the mixed sugars, xylose and glucose were consumed after 3 and 2 days, respectively, of cultivation and a residual glycerol amount of 5.3 g L^−1^ was observed after 7 days of cultivation (Figure 5c). The maximum yield of TAGs per gram of carbon source consumed during 7 days of cultivation was 0.136 (±0.006) g g^−1^ on the xylose/glucose/glycerol mix, and 0.151 (±0.004) g g^−1^ on the xylose/glucose mix with pulse loading glycerol. When glycerol in the xylose/glucose mix medium was added, the maximum TAG yield per carbon source consumed was higher than that with the xylose/glucose mix alone. The identity of the lipids and the fatty acid composition of MITGX-173 grown under those conditions were similar to one another. The major component of the intracellular lipids was TAGs, and the fatty acids consisted primarily of palmitic acid (26 to 29%), *cis*-10-heptadecenoic acid (18 to 21%) and oleic acid (13 to 15%) (Figure 5d, e).

## Discussion

The capability to utilize glycerol efficiently is advantageous for microbial conversion process to develop cost-effective, streamlined bioprocesses in renewable fuel production. In addition, development of microbial technology for efficiently converting lignocellulosic biomass to biofuels has been considered as a potential solution for reducing current petroleum consumption and carbon emissions. Previously, we have developed a TAG-producing *R. opacus* MITXM-61 strain, which can efficiently coferment with glucose and xylose present predominantly in hydrolysates of lignocellulosic biomass [54]. Strain MITXM-61 was able to grow faintly on glycerol. The use of evolutionary engineering has proven to be instrumental in obtaining phenotypes of microorganisms with improved properties [58-60]. In present work, therefore, we applied an evolutionary approach to improve glycerol utilization in the *R. opacus*.

As a result, we constructed a TAG-producing *R. opacus* MITGM-173 strain capable of growing on glycerol concentrations greater than 150 g L^−1^ (Figure 1), as well as xylose. The strain grown on a mixture of 40 g L^−1^ glucose, 40 g L^−1^ xylose and 20 g L^−1^ glycerol utilized the substrates at maximum consumption rates of 0.833 g L^−1^ h^−1^, 0.721 g L^−1^ h^−1^ and 0.119 g L^−1^ h^−1^, respectively, and yielded 13.6 g L^−1^ of TAGs after 7 days of cultivation (Figure 5b). Strain MITGM-173 was capable of completely utilizing carbon sources in a 2:2:1 ratio of glucose/xylose/glycerol with an initial total substrate concentration of 100 g L^−1^. The maximum TAG production of MITGM-173 grown on a mixture of 40 g L^−1^ glucose and 40 g L^−1^ xylose was 10.2 g L^−1^ with maximum consumption rates of 0.923 g L^−1^ h^−1^ by glucose, and 0.843 g L^−1^ h^−1^ by xylose (Figure 5a). The TAG yield per substrate consumed (0.136 g g^−1^) on a 2:2:1 ratio of glucose/xylose/glycerol was slightly higher than that (0.128 g g^−1^) observed on the 2:2 glucose/xylose without glycerol. The consumption curve of three substrates on the 2:2:1 glucose/xylose/glycerol culture sequentially exhibited a simultaneous pattern in contrast to a diauxie. Most microorganisms preferentially consume glucose in mixed substrates due to carbon catabolite repression or allosteric competition of the sugar in sugar transport [61,62]. The diauxic metabolism has been a major bottleneck to complete conversion and efficient utilization of multiple substrates [63]. Strain MITGM-173 was capable of utilizing multiple substrates independent of each other, although the consumption rate on glycerol was remarkably slower than that on glucose or xylose.

In addition, cultivating in a mixture of 40 g L^−1^ xylose and 40 g L^−1^ glucose with pulse loading of 20 g L^−1^ glycerol after 2 days of cultivation, MITGM-173 consumed the pulsed glycerol at the maximum consumption rate of 0.123 g L^−1^ h^−1^ and produced 14.3 g L^−1^ of TAGs corresponding to 0.151 g g^−1^ yield per carbon sources consumed after 7 days of cultivation, at which point the observed residual glycerol concentration was 5.3 g L^−1^ (Figure 5c). Interestingly, the maximum TAG production and the TAG yield per carbon sources consumed in the mixture with the pulse loading of glycerol were higher than those with the initial addition of glycerol. Based on the results obtained in mixtures containing 40 g L^−1^ glucose and 40 g L^−1^ xylose (Figure 5a), excessive addition of 20 g L^−1^ glycerol in the glucose/xylose resulted in an increase of 3.4 g L^−1^ TAGs (no residual glycerol) on the initial addition, and 4.1 g L^−1^ (residual glycerol of 5.3 g/L) on the pulse loading, corresponding to 0.170 g g^−1^ and 0.279 g g^−1^, respectively, of the TAG yield per glycerol consumed (Figure 5b, c). The results showed that the pulse loading is a better approach compared to initial addition in regard to improvement of TAG productivity on glycerol in the *R. opacus.* The pulsed glycerol might serve as the direct intracellular precursor for synthesis of TAGs. More importantly, elucidation of this unique glycerol-assimilation mechanism should be of great importance in developing a superior strain, which results in a high yield of TAGs. The maximum theoretical conversion of glucose to TAGs in microorganisms is approximately 0.316 g g^−1^ (TAG yield per sugar consumed) [64]. One of the major challenges for developing the cost-effective streamlined bioprocess is to achieve yields that are close to theoretical limits [65].

Thus, the fermentation performance of strain MITGM-173 on glycerol may provide important clues to the construction of an industrial strain with high TAG yield. While the simultaneous and complete utilization of multiple substrates of strain MITGM-173 is ideal for production from renewable resources, the very slow consumption of glycerol would seem to be a barrier to its industrial application. The relatively shorter consumption by *R. opacus* on multiple substrates simultaneously could make this organism a practical bioconverter for TAG production. Glycerol metabolism by *Rhodococcus* species has not been studied thoroughly. Further studies need to be carried out to elucidate the genetic changes involved in the improvement of glycerol utilization and the underlying glycerol assimilation mechanism as well as the mechanism and regulation of simultaneous metabolism of multiple carbon substrates by *R. opacus*. The present study demonstrated that an evolved strain, MITGM-173, has potential towards production of advanced biofuels from low-cost feedstocks of glycerol and lignocellulosic biomass.

## Conclusions

The adaptive evolution approach has proved useful for optimization of the inefficient glycerol-utilizing *R. opacus* strain. The evolved strain, MITGM-173, shows significantly improved glycerol metabolizing capacity in comparison to the parental strain, MITXM-61. Strain MITGM-173 was capable of utilizing high concentrations of glycerol or mixed glycerol/glucose/xylose simultaneously and producing large amounts of TAGs. The pulse addition of glycerol into the glucose/xylose mixture resulted in conspicuously increased TAG yield per glycerol consumed. Elucidation of the underlying glycerol-assimilation mechanism will provide great potential in constructing an industrial strain with high TAG yield. This study revealed that the evolved *R. opacus* strain has critical features of the biocatalyst for developing a cost-effective manufacturing paradigm to generate advanced renewable fuels. To our knowledge, this is the first report of any *Rhodococcus* strains capable of cofermenting glycerol, glucose and xylose.

## Methods

### Bacterial strains and media

A list of strains used in this study is given in Additional file 2: Table S1. *R. opacus* MITXM-61 was constructed in our previous study [54]. Glycerol-fermenting MITXM-61-derivatives were constructed in this study. The culture media used were LB broth (BD Diagnostic Systems, Sparks, MD) and a defined medium containing the following composition per liter: 16 g glycerol, 1.0 g (NH_4_)_2_SO_4_, and mineral components consisted of 1.0 g MgSO_4_•7H_2_O, 0.015 g CaCl_2_•2H_2_O, 1.0 ml of a trace element solution, 1.0 ml stock A solution, and 35.2 ml 1.0 M phosphate buffer as described [66]. Modifications of the defined medium are stated in table and figure legends. Solid media were supplemented with 2% (*w*/*v*) agar. The strains were routinely maintained on LB agar medium and preserved in 20% (*v*/*v*) glycerol at −80 °C. All chemicals were obtained from Sigma-Aldrich (St. Louis, MO) unless otherwise noted.

### Strain construction

Competent cells of *R. opacus* MITXM-61 were prepared, as previously described [54], and treated with electroporation (Bio-Rad gene pulser, Hercules, CA) at 2.5 kV, 25 μF, and 200 Ω in a 2-mm electroporation cuvette (VWR, Radnor, PA). The pulsed cells were diluted with LB broth, regenerated for 3 h with gentle agitation, plated onto a defined agar medium containing 16 g L^−1^ glycerol, and incubated to harbor glycerol utilizing strains. After 10 days of cultivation, spontaneous mutants that exhibited robust growth on glycerol were isolated.

Strain MITGM-73, one of the glycerol-utilizing isolates, was used for serial transfers of cells using repetitive cultures in flasks. The cells grown on LB agar medium for 3 days were inoculated into a 250-mL baffled flask with 50 mL of the defined medium containing 100 g L^−1^ glycerol to an initial OD of 1.0. When the cells were grown to early stationary phase, 5 mL of culture broth from the preceding flask was transferred to a new flask culture with the same medium composition. This procedure was repeated for four iterations. From the culture broth after a total of 4 generations (22 days), 20 colonies were randomly isolated by plating for single clones on a defined agar medium with 16 g L^−1^ glycerol and tested again for growth in flasks with a defined medium containing 100 g L^−1^ glycerol. One of the fastest growing strains was named MITGM-173 and used for further experiments.

### Fermentation conditions

All cultures were grown at 30°C. Cell growth was monitored by determining the optical density (OD) at 660 nm (Thermo Scientific GENESYS 20, Waltham, MA). *R. opacus* seed cultures were prepared in a modified defined medium supplemented with 16 g L^−1^ glucose. Cells from colonies grown on LB agar medium for 3 days were inoculated into the modified medium in a flask. The culture was cultivated for 2 days until the late exponential phase. Unless otherwise stated, cultures for flask and bioreactor experiments were inoculated with the seed culture to an initial OD of 1.0 (2.5 × 10^8^ cfu mL^−1^). Shake flask experiments were carried out using 250-mL baffled flasks with a working volume of 50 mL and incubated on a rotary shaker at 200 rpm (Multitron, Infors, Bottmingen, Switzerland). Bioreactor experiments were conducted in a 2-L fermentor (Bioengineering bioreactor, R’ALF, Wald, Switzerland) with a working volume of 1 L. The pH of the medium was kept constant at 6.9 ± 0.1 by the automatic addition of 2 M NaOH. The dissolved oxygen level was monitored using an Ingold polarographic probe (Mettler-Toledo Ingold Inc., Bedford, MA). The dissolved oxygen tension was maintained above 60% by using an adjusted stirrer profile for increasing the agitation speed from 300 rpm up to 1,000 rpm and automatically sparging with a mixture of air and pure oxygen at a constant gas flow rate of 1.0 vvm. When necessary, polypropylene glycol P 2,000 was manually added to each vessel to prevent foam formation.

### Response surface methodology for optimization of TAG production

The ratio of carbon to nitrogen (C/N) in the medium is the most important for increased TAG production in *R. opacus* [51,52]. The statistical experimental approach using Box-Wilson central composite design [67] with five settings for each of two factors was applied in the optimization of TAG production from glycerol. Glycerol concentration (g L^−1^) and (NH_4_)_2_SO_4_ concentration (g L^−1^) were chosen as independent variables, and TAG production (g L^−1^) was used as a dependent output variable. A set of 11 runs was conducted with nine combinations of glycerol and (NH_4_)_2_SO_4_ concentrations including three repetitions at the central point in submerged batch fermentations (Table 1). The software StatGraphics (StatPoint Inc., USA) was used for regression and graphical analysis of the data. The optimum values of the selected variables were obtained by solving the regression equation. Three-dimensional surface plots were drawn by SigmaPlot 11 (Systat Software, Inc., San Jose, CA) to display the interaction among various variables.

### Analytical methods

CDW was determined by lyophilizing cell pellet after centrifuging 10 mL of culture broth at 8,000 g for 15 min and washing the cell pellet twice in deionized water. The lyophilized cell pellet was used to analyze the identity of lipids and the fatty acid composition. For the identification of lipids, TLC experiments were carried out using a two-step resolution method as previously described [51]. Lyophilized cell pellets were extracted with methanol and chloroform (1:1, *v*/*v*) and incubated at room temperature for 1 h with gentle agitation. Ten micrograms of crude lipid extract was spotted onto silica gel 60 plates (EMD Chemicals Inc., Gibbstown, NJ). Samples were resolved using an initial polar solvent system consisting of 60:35:5 chloroform/methanol/water, followed by a second solvent system containing 70:30:1 hexane/diethyl ether/acetic acid. Resolved lipids were visualized by charring. Plates were sprayed with a 3% cupric acetate-8% aqueous phosphoric acid solution followed by baking in a 200°C oven for 5 min. To determine the fatty acid content of the cells and the composition of lipids, the whole cells were subjected to methanolysis and the resulting fatty acid methyl esters (FAMEs) were analyzed by GC as described in detail recently [51,53]. GC analysis of FAMEs was performed by using an Agilent 6850 series II network GC system equipped with an Agilent DB-Wax column (30 m by 0.32 mm, 0.5 μm film) (Agilent Technologies, Santa Clara, CA) with hydrogen as the carrier gas. A 2-μL portion of the sample was injected with a 30:1 split ratio. The inlet was maintained at 250°C. The oven was held at 80°C for 5 min, heated to 220°C at 20°C min^−1^, and then held at 220°C for 5 min. Peak detection was carried out by a flame ionization detector, which was maintained at 300°C. The fatty acids were identified and quantified by comparison to standard FAMEs. Fatty acid content was defined as the percentage of the ratio of fatty acids to cell dry weight (% CDW). Total lipid content was calculated as the sum of total fatty acid contents for nine FAMEs: methyl myristate (C14:0), methyl pentadecanoate (C15:0), methyl palmitate (C16:0), methyl palmitoleate (C16:1), methyl heptadecanoate (C17:0), methyl *cis*-10-heptadecenoate (C17:1), methyl stearate (C18:0), methyl oleate (C18:1), and methy linoleate (C18:2). The supernatants of the culture broth were used for analyses of residual glycerol, glucose, xylose, and (NH_4_)_2_SO_4_ after filtration through 0.2-μm syringe filters. Glycerol, glucose, and xylose concentrations in the culture were measured by high-performance liquid chromatography (HPLC; Agilent 1100 system) fitted with an Aminex HPX-87H column (300 × 7.8 mm, Bio-Rad) coupled to a refractive index (RI) detector as previously described [51,53]. Ammonia concentration in the culture was measured using a Sigma Ammonia Assay Kit according to the manufacturer’s instructions.

## Additional files

Additional file 1: Figure S1. Glycerol-utilizing colonies of *R. opacus* on plates. Cells of a xylose-fermenting MITXM-61 strain after electroporation were spread on a defined agar medium containing 16 g L^−1^ glycerol and incubated for 10 days. **Figure S2.** Growth of *R. opacus* MITXM-61 derivatives on glycerol. Each strain was grown in a defined medium containing 16 g L^−1^ glycerol in shake flasks. Values and error bars represent the mean and s.d. of triplicate experiments. **Figure S3.** Adaptive evolution of *R. opacus* MITGM-73 for improved glycerol utilization. The strain was grown in a modified defined medium containing 100 g L^−1^ glycerol in a flask. Five milliliters of the culture were sequentially transferred into a flask containing 50 mL of the fresh modified medium after 6, 10, 14, and 18 days of cultivation as indicated by the arrow. **Figure S4.** Time course kinetics of TAG production as fatty acids from glycerol and/or glucose by *R. opacus* MITXM-61. The strain was grown in defined media containing 16 g L^−1^ glycerol (a), a mixture of 8 g L^−1^ glycerol and 8 g L^−1^ glucose (b), and 16 g L^−1^ glucose (c) in shake flasks. Values and error bars represent the mean and s.d. of triplicate experiments. Additional file 2: Table S1. Bacterial strains used in this study.

## Abbreviations

TAG: Triacylglycerol
CDW: Cell dry weight
OD: Optical density
C/N: Carbon to nitrogen ratio
HPLC: High-performance liquid chromatography
GC: Gas chromatography
FAME: Fatty acid methyl ester
TLC: Thin-layer chromatography
