# Supplementary material for: Improved glycerol utilization by a triacylglycerol-producing Rhodococcus opacus strain for renewable fuels
Source: Biotechnol Biofuels. 2015 Feb 26;8:31. doi: 10.1186/s13068-015-0209-z (PMC4355421; doi:10.1186/s13068-015-0209-z)
Supplement: Additional file 1: Figure S1. — Glycerol-utilizing colonies of R. opacus on plates. Cells of a xylose-fermenting MITXM-61 strain after electroporation were spread on a defined agar medium containing 16 g L−1 glycerol and incubated for 10 days. Figure S2. Growth of R. opacus MITXM-61 derivatives on glycerol. Each strain was grown in a defined medium containing 16 g L−1 glycerol in shake flasks. Values and error bars represent the mean and s.d. of triplicate experiments. Figure S3. Adaptive evolution of R. opacus MITGM-73 for improved glycerol utilization. The strain was grown in a modified defined medium containing 100 g L−1 glycerol in a flask. Five milliliters of the culture were sequentially transferred into a flask containing 50 mL of the fresh modified medium after 6, 10, 14, and 18 days of cultivation as indicated by the arrow. Figure S4. Time course kinetics of TAG production as fatty acids from glycerol and/or glucose by R. opacus MITXM-61. The strain was grown in defined media containing 16 g L−1 glycerol (a), a mixture of 8 g L−1 glycerol and 8 g L−1 glucose (b), and 16 g L−1 glucose (c) in shake flasks. Values and error bars represent the mean and s.d. of triplicate experiments. [file 13068_2015_209_MOESM1_ESM.pdf]

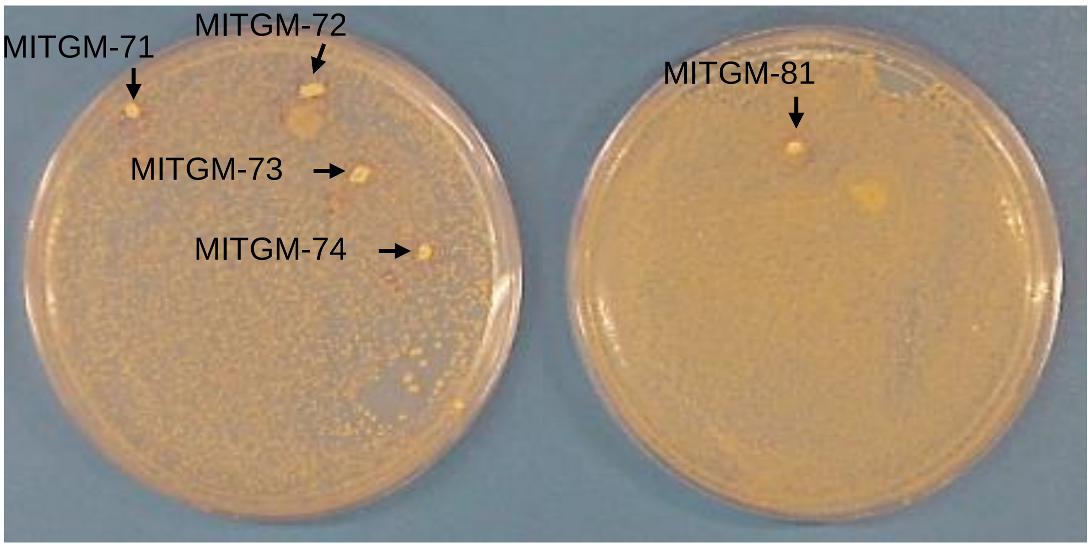

Figure S1 Glycerol-utilizing colonies of *R. opacus* on plates. Cells of a xylose-fermenting MITXM-61 strain after electroporation were spread on a defined agar medium containing 16 g L^{-1} glycerol, and incubated for 10 days.

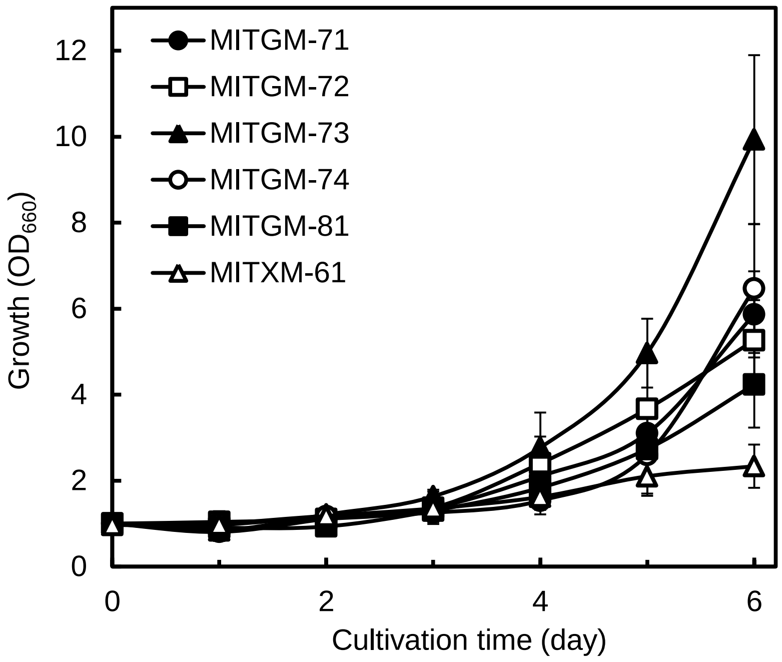

Figure S2 Growth of *R. opacus* MITXM-61-derivatives on glycerol. Each strain was grown in a defined medium containing 16 g L^{-1} glycerol in shake flasks. Values and error bars represent the mean and s.d. of triplicate experiments.

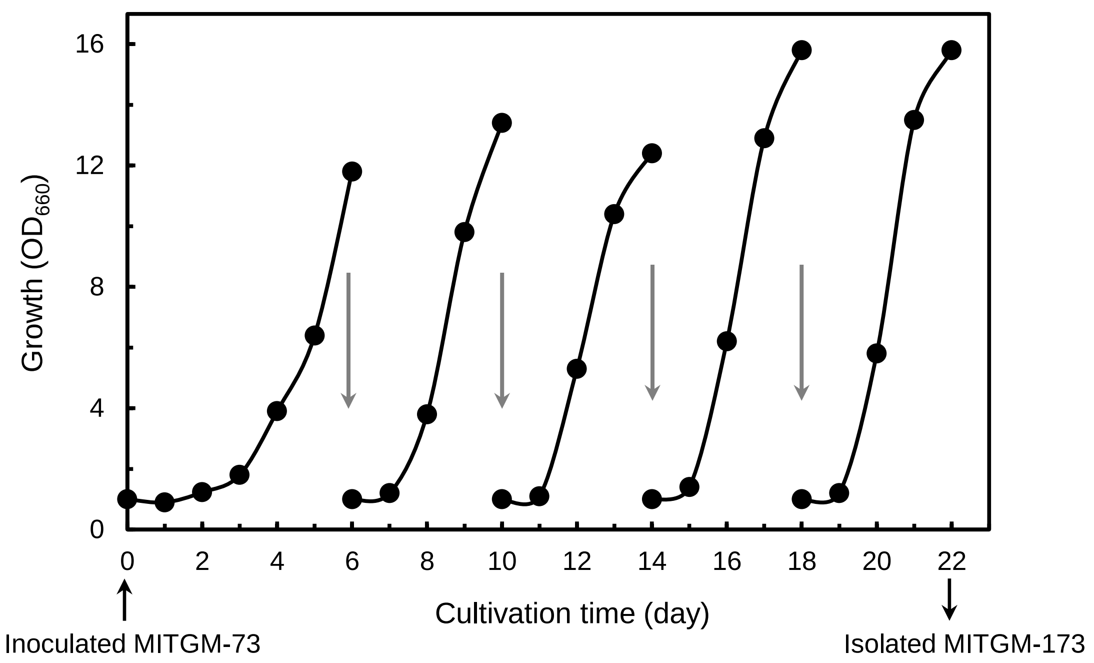

Figure S3 Adaptive evolution of *R. opacus* MITGM-73 for improved glycerol utilization. The strain was grown in a modified defined medium containing 100 g L^{-1} glycerol in a flask. Five milliliters of the culture were sequentially transferred into a flask containing 50 ml of the fresh modified medium after 6, 10, 14 and 18 days of cultivation as indicated by the arrow.

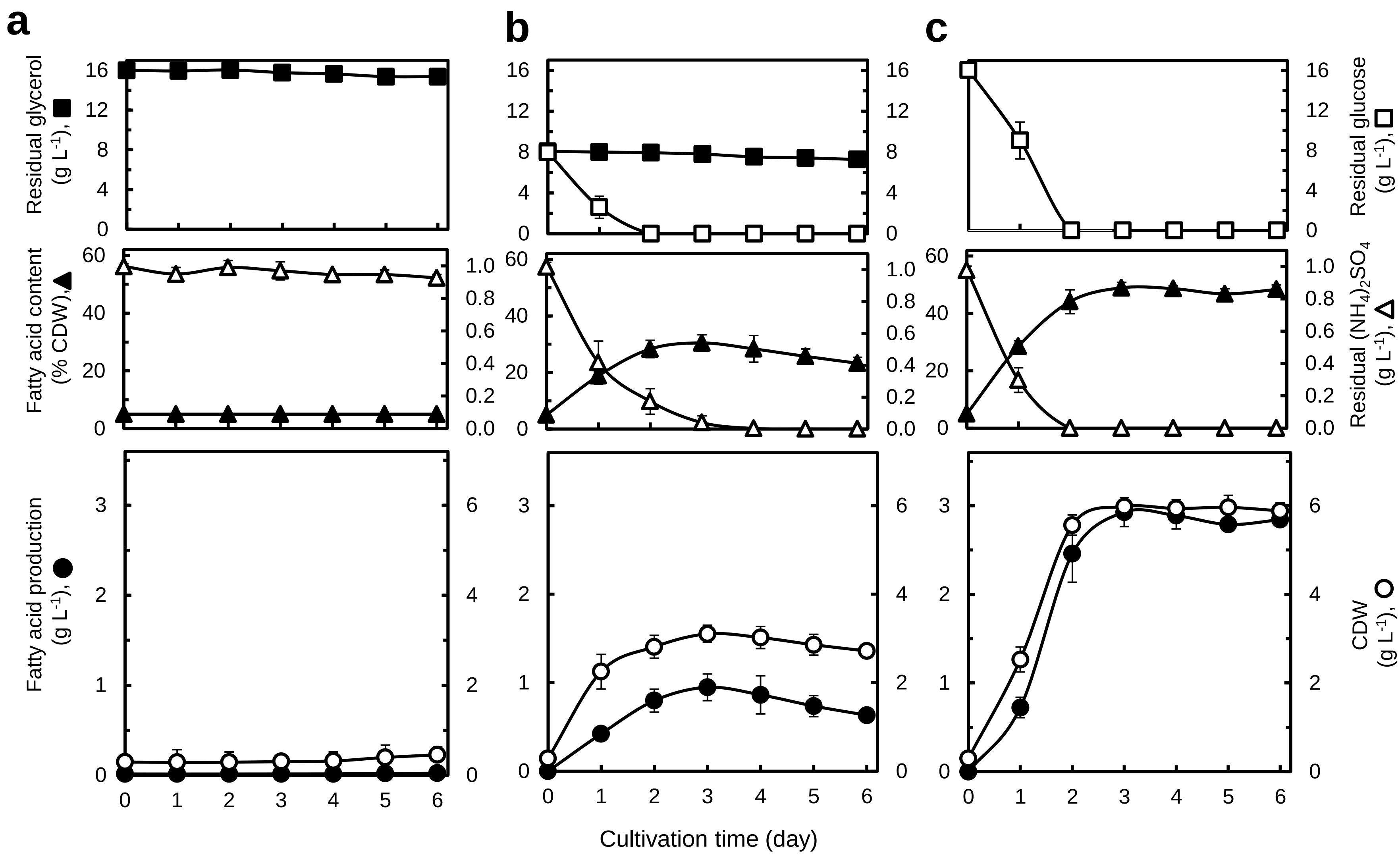

Figure S4 Time course kinetics of TAG production as fatty acids from glycerol and/or glucose by *R. opacus* MITXM-61. The strain was grown in defined media containing 16 g L^{-1} glycerol **(a)**, a mixture of 8 g L^{-1} glycerol and 8 g L^{-1} glucose **(b)**, and 16 g L^{-1} glucose **(c)** in shake flasks. Values and error bars represent the mean and s.d. of triplicate experiments.
