# Supplementary material for: Improved glycerol utilization by a triacylglycerol-producing Rhodococcus opacus strain for renewable fuels
Source: Biotechnol Biofuels. 2015 Feb 26;8:31. doi: 10.1186/s13068-015-0209-z (PMC4355421; doi:10.1186/s13068-015-0209-z)
Supplement: Additional file 2: Table S1. — Bacterial strains used in this study. [file 13068_2015_209_MOESM2_ESM.pdf]

Table S1 Bacterial strains used in this study

Strain	Description	Reference
Rhodococcus opacus		
MITXM-61	Mutant evolved from Xsp8C	[54]
MITGM-71	Mutant evolved from MITXM61	This study
MITGM-72	Mutant evolved from MITXM61	This study
MITGM-73	Mutant evolved from MITXM61	This study
MITGM-74	Mutant evolved from MITXM61	This study
MITGM-81	Mutant evolved from MITXM61	This study
MITGM-173	Mutant evolved from MITGM-73	This study
